# Green for us: parental compensation for children’s unsustainable behaviors

**DOI:** 10.3389/fpsyg.2024.1529563

**Published:** 2025-01-14

**Authors:** Sili Wang, Xiaofei Zhang

**Affiliations:** ^1^School of Economics and Management (School of Tourism), Dalian University, Dalian, Liaoning, China; ^2^School of Management, Northeastern University at Qinhuangdao, Qinhuangdao, Hebei, China

**Keywords:** sustainable behaviors, children’s unsustainable behaviors, family responsibility, environmental responsibility, intergenerational caregiving, parental caregiving

## Abstract

This study explores the impact of children’s unsustainable behaviors on parental sustainable actions within family dynamics. Findings reveal that parents exposed to their children’s unsustainable behaviors experience heightened family and environmental responsibility, which motivates them to engage in both private-domain and public-domain sustainable behaviors. These effects are amplified in intergenerational caregiving contexts, where parents compensate for reduced caregiving roles by adopting more sustainable practices. Through four experiments, the research validates the mediating roles of family and environmental responsibility and the moderating influence of caregiver type. This study extends existing theories on intergenerational behavior transmission by highlighting the influence of children’s unsustainable behaviors, offering valuable insights for family education strategies and policy development aimed at fostering sustainability within households.

## 1 Introduction

The escalating severity of global environmental challenges has underscored the critical importance of promoting sustainable behaviors. While public awareness and concern regarding environmental issues have grown, this heightened awareness has not consistently translated into concrete behavioral changes in daily life (Voltmer and von Salisch, 2023). Studies suggest that unsustainable behaviors within society are significantly shaped by group dynamics and social norms, with individuals often yielding to non-eco-friendly consumption patterns under the influence of social pressure (Žukauskienė et al., 2020). For example, research has examined how varying social groups, including strangers (Schultz et al., 2007), temporary group members, and close acquaintances such as friends and colleagues (Meijers et al., 2019), influence individual participation in sustainable practices (Cakanlar et al., 2023). Evidence indicates that exposure to unsustainable behaviors by neighbors and others frequently encourages the adoption of similar behaviors, rather than motivating individuals to offset such behaviors through their own sustainable actions (Meijers et al., 2019; Cakanlar et al., 2023). Moreover, the widespread use of social media and digital platforms has accelerated the dissemination of unsustainable behaviors, further entrenching non-eco-friendly practices as social norms (Alrusaini and Beyari, 2022).

The influence of close family members on one another differs markedly from other social influences, as it often arises from a shared sense of familial identity (Rowe, 1995), wherein individuals perceive family members as extensions of themselves. For instance, research on marital relationships by (Cakanlar et al., 2023) demonstrated that when one partner observed unsustainable behaviors in their spouse, those with greater relational power were more likely to take responsibility and engage in sustainable behaviors to reinforce their shared marital identity.

In the context of intergenerational relationships, studies have shown that parents play a pivotal role in shaping their children’s sustainable behaviors through mechanisms such as modeling, parent–child communication, and parent–child participation (Li et al., 2023). Conversely, children of various age groups can also influence their parents’ adoption of sustainable practices. For example, many parents report being influenced by the environmentally conscious actions requested by children aged 3–7, actions often derived from knowledge acquired through eco-school programs (Spiteri, 2020). Additionally, research highlights that adolescents not only absorb environmental values imparted by their parents but also actively shape parental behaviors, particularly in domains such as reducing plastic usage and promoting recycling efforts (Žukauskienė et al., 2020). These findings underscore the significant role of children in fostering sustainability within the family unit.

In recent years, scholars have increasingly recognized the critical role of families as “primary consumption units” (Bulkeley and Gregson, 2009) in driving sustainable development. As significant members of the family unit, children play an essential role in this process. Existing research has primarily focused on the mechanisms of intergenerational transmission of sustainable behaviors between parents and children. However, limited attention has been given to the influence of children’s unsustainable behaviors (e.g., wasteful practices) on family interactions. To address this gap, the present study aims to explore the intergenerational dynamics of sustainable behaviors within families triggered by children’s unsustainable actions, highlighting their potential impact on both household and societal sustainability practices.

Building on this foundation, this study employs four experiments to systematically investigate the impact of children’s unsustainable behaviors on parents’ sustainable behaviors and the underlying mechanisms. The core research questions addressed in this study include the following:

1. Does children’s unsustainable behavior influence parents’ sustainable behavior?
2. Do family responsibility and environmental responsibility mediate the relationship between children’s unsustainable behaviors and parents’ sustainable behaviors?
3. Does caregiver type (e.g., intergenerational caregiving vs. parental caregiving) moderate the above relationships?

Theoretically, this study expands the research framework on the impact of family member interactions on sustainable behaviors by introducing the perspective of children’s unsustainable behaviors, offering a novel lens for understanding family dynamics in the context of sustainability. Additionally, it validates the moderating role of caregiver type, providing theoretical insights and boundary condition analyses for sustainable behaviors across different family structures. Practically, this study offers a scientific basis for designing family-based environmental education strategies by enhancing parental responsibility to effectively address children’s unsustainable behaviors.

## 2 Conceptual framework and research hypotheses

### 2.1 Children’s unsustainable behaviors and parents’ sustainable behaviors

Public-domain sustainable behaviors refer to actions that have broad societal and environmental impacts, typically occurring in public settings or during interactions with others. Examples include participating in collective environmental initiatives or supporting eco-friendly policies. In contrast, private-domain sustainable behaviors primarily pertain to individual or family-oriented actions, often carried out in private settings, such as conserving energy or reducing waste (Mateer et al., 2022; Stern, 2000).

Within the family context, the sharing of resources and alignment of goals between parents and children form a complex dynamic of interactions (Hasford et al., 2018). The influence of children’s behavior on parents differs from other social influences, as parents often integrate caregiving roles into their self-concept (Acitelli et al., 1999), fostering a sense of family identity. This family identity plays a crucial role in how parents respond to their children’s behaviors, particularly unsustainable ones, such as wasteful practices (Walsh and Neff, 2018). According to Emotional Reaction Theory, an individual’s emotional experiences influence their behavioral decisions, particularly when confronted with negative behaviors involving significant others (Lazarus, 1991). When parents encounter their children’s unsustainable behaviors, they often experience emotional reactions, such as guilt or concerns about their children’s future quality of life (Fahim et al., 2021). These emotional responses may motivate parents to adopt more sustainable lifestyles, driven not only by cognitive decision-making but also by a direct concern for the wellbeing of family members (Zhao et al., 2020).

Moreover, when parents engage in behaviors related to their children, they are more likely to adopt actions aligned with social norms and ethical standards. For instance, they may reduce environmentally harmful consumption patterns. Such sustainable behaviors are not merely performative but are rooted in parents’ deep sense of family responsibility and considerations for their children’s future (Collins, 2015). Consequently, when addressing their children’s unsustainable behaviors, parents are more inclined to engage in both private- and public-domain sustainable behaviors.

*H1a:* Parents are more likely to engage in private-domain sustainable behaviors after being exposed to their children’s unsustainable behaviors, as compared with their baseline tendencies (i.e., no exposure to their children’s behaviors).

*H1b:* Parents are more likely to engage in public-domain sustainable behaviors after being exposed to their children’s unsustainable behaviors, as compared with their baseline tendencies (i.e., no exposure to their children’s behaviors).

### 2.2 The mediating role of family responsibility and environmental responsibility

Family responsibility refers to an individual’s awareness of their obligations and duties within the family (Kim and Kochanska, 2020a), which is an integral component of the broader system of civic responsibility. Role Theory posits that individuals assume specific role responsibilities within particular social contexts and adjust their behavior according to the demands of these roles (Biddle, 1986). Interactions between parents and children often prompt parents to adjust their behaviors to meet their children’s developmental needs (Qin and Pomerantz, 2013). During these interactions, parents are influenced by their children’s behaviors, which can evoke a strong sense of family responsibility and lead to behavioral adjustments. For instance, when children face learning difficulties or encounter challenges in school, parents often experience an enhanced sense of family responsibility and become more actively involved in their children’s education and learning support (Kim and Kochanska, 2020a). In such situations, children’s academic challenges act as signals that prompt parents to recognize their roles and responsibilities. This recognition motivates them to take actions such as communicating with teachers, providing additional learning resources, or adjusting family routines to support their children’s learning (Curelaru et al., 2020). Similarly, when children exhibit shyness or lack social skills in social settings, parents often feel responsible for helping their children improve these skills. Parents may create more social opportunities for their children or practice social interaction techniques with them to help them better adapt to social environments (Lin, 2023).

The Norm Activation Model (NAM), proposed by Schwartz (1977), emphasizes that the activation of personal moral norms motivates individuals to engage in prosocial behaviors, including sustainable behaviors. Environmental responsibility is a core component of moral norm activation. When parents observe their children’s unsustainable behaviors, they may develop a heightened sense of environmental moral responsibility, becoming more aware of the potential impacts of their actions on the environment. This moral drive encourages parents to fulfill their environmental responsibilities through sustainable behaviors, such as participating in environmental initiatives or reducing resource waste (Lee et al., 2022).

When confronted with children’s unsustainable behaviors, parents often interpret these behaviors as signals indicating potential shortcomings in family environmental education or resource usage (Jia and Yu, 2021). These behavioral signals can trigger a heightened sense of family responsibility, motivating parents to take corrective or compensatory actions. For example, when children engage in wasteful or environmentally harmful behaviors, parents are more likely to adopt proactive eco-friendly actions, such as reducing household energy consumption or paying closer attention to waste sorting (Lawson et al., 2019). These compensatory behaviors not only directly address children’s unsustainable actions but also serve as a way for parents to model positive behaviors within the family and convey pro-environmental values (Žukauskienė et al., 2020). Through this process, parents aim to influence their children’s behaviors while demonstrating their commitment to environmental responsibility, guiding the family as a whole toward greater sustainability.

Thus, family responsibility plays a pivotal mediating role when parents confront children’s unsustainable behaviors. By stimulating parents’ self-regulatory behaviors, it drives them to adopt sustainable lifestyles. This behavioral adjustment not only fulfills the demands of family education and role modeling but also reflects parents’ intrinsic recognition and practice of their family responsibilities.

*H2a:* Family responsibility mediates the relationship between children’s unsustainable behaviors and parents’ private-domain sustainable behaviors.

*H2b:* Family responsibility mediates the relationship between children’s unsustainable behaviors and parents’ public-domain sustainable behaviors.

As individuals, parents generally have an existing awareness of environmental issues, such as climate change and resource waste. When they realize that their children’s behaviors may exacerbate these problems, it often triggers environmental concerns and strengthens their sense of environmental responsibility (Sun et al., 2020). Parents believe that even small changes in their behavior can have a positive impact on the environment. Consequently, after observing their children’s behaviors, parents often choose to engage in sustainable practices as a response. These actions not only serve as role models for their children but are also perceived by parents as effective ways to contribute to tangible environmental improvements. Relevant research has demonstrated a significant association between self-efficacy and positive changes in pro-environmental behaviors (Lee et al., 2022).

As parents, individuals often experience a heightened awareness of certain environmental beliefs following the birth of their children (Milfont et al., 2020). Thus, when parents notice their children’s unsustainable behaviors, it often prompts them to pay greater attention to the state of their living environment and the future conditions for their descendants. This heightened sense of responsibility further motivates them to adopt sustainable practices that contribute to environmental wellbeing. Driven by this sense of responsibility, parents actively engage in behaviors that aim to benefit the environment.

*H3a:* Environmental responsibility mediates the relationship between children’s behaviors and parents’ private-domain sustainable behaviors.

*H3b:* Environmental responsibility mediates the relationship between children’s behaviors and parents’ public-domain sustainable behaviors.

### 2.3 The moderating role of caregiver type

In this study, “caregiver type” is defined as the role of the primary individual responsible for the care and education of children within the family. It primarily includes parental caregiving and intergenerational caregiving. Parental caregiving refers to parents serving as the primary caregivers, engaging directly in the daily interactions and education of their children. Intergenerational caregiving refers to grandparents assuming the primary caregiving role, taking responsibility for daily care and education.

The Identity-Based Motivation Theory posits that human motivation and goal pursuit are context-dependent, with an individual’s identity or self-concept driving them to take action to achieve their goals in different situations. Specifically, individuals tend to regulate their behavior to strive toward their desired future identity and act in ways consistent with both their current and aspirational identities (Oyserman, 2013; Oyserman and Dawson, 2021). When caregivers are intergenerational caregiving (i.e., grandparents), parents often have less direct involvement in their children’s care, which may lead to feelings of guilt. These emotions encourage compensatory behaviors, where parents believe they should invest more effort in their children, thereby cultivating a stronger sense of family responsibility. Driven by this responsibility, parents are more likely to engage actively with their children or improve their parenting roles to bridge emotional gaps with their children. Research suggests that parents who lack direct caregiving roles often pay closer attention to their children’s behavior and feel a responsibility to educate their children through role modeling (Harris et al., 2020). When parents reestablish contact with their children, they often focus more on cultivating behavioral norms and healthy habits, particularly in emotional wellbeing and sustainable behavior development. This compensatory behavior reflects parents’ desire to serve as positive role models for their children, especially in areas of sustainability and responsibility, emphasizing health, education, and moral guidance. Such behavior is often a manifestation of compensatory family responsibility stemming from a lack of daily caregiving (Zhou et al., 2018).

In contrast, in intragenerational caregiving contexts, where parents are directly responsible for raising their children, interactions between parents and children are typically more frequent and direct, reducing feelings of caregiving deficits (Zhou et al., 2018). This frequent interaction prompts parents to actively manage their children’s behavior and provide timely feedback on inappropriate actions, often through methods such as explanation or reasoning to correct the behavior (Pöyhönen et al., 2020). However, when confronted with children’s unsustainable behaviors, parents in this context may be more likely to justify their children’s actions, for example, by saying, “The child is too young,” or attributing the behavior to the child’s inherent traits. This can lead to shifting responsibility and passive responses, such as “I already told you” (Gershoff et al., 2012). As a result, in intragenerational caregiving settings, the impact of children’s unsustainable behaviors on parents’ sustainable actions through family responsibility is generally weaker.

*H4:* Caregiver type moderates the relationship between children’s unsustainable behaviors and family responsibility.

*H5a:* Caregiver type moderates the mediating effect of family responsibility on the relationship between children’s behaviors and parents’ private-domain sustainable behaviors. Compared to intragenerational caregiving, family responsibility plays a more significant mediating role in intergenerational caregiving contexts.

*H5b:* Caregiver type moderates the mediating effect of family responsibility on the relationship between children’s behaviors and parents’ public-domain sustainable behaviors. Compared to intragenerational caregiving, family responsibility plays a more significant mediating role in intergenerational caregiving contexts.

When children are raised by grandparents, interactions between parents and children are significantly reduced, making it difficult for parents to provide real-time feedback and management of their children’s behaviors. In such situations, parents often fail to correct inappropriate behaviors in a timely manner (Li et al., 2022), particularly in areas related to environmental sustainability. Observing their children’s unsustainable behaviors typically triggers parental concerns about the future of the environment, further enhancing their sense of environmental responsibility. This heightened responsibility is often rooted in deep concerns for their children’s future quality of life and the overall health of the global environment (Herzog et al., 2021). Intergenerational caregiving inherently means that parents are not directly involved in managing their children’s daily behaviors. This “absence” not only leads to a perceived loss of control over their children’s education but also exacerbates parents’ anxiety about environmental issues.

In contrast, when parents are directly involved in caregiving, they are able to guide their children’s behaviors more effectively through real-time feedback mechanisms. For example, when children display unsustainable behaviors, parents can intervene promptly (Pöyhönen et al., 2020). Such direct interventions not only help to improve children’s behaviors but also alleviate parents’ concerns and anxieties about future environmental challenges. Through timely and direct feedback, parents can translate their sense of environmental responsibility into specific educational actions, thereby mitigating their anxieties about environmental degradation to some extent.

*H6*: Caregiver type moderates the relationship between children’s unsustainable behaviors and parental environmental responsibility.

*H7a:* Caregiver type moderates the mediating effect of environmental responsibility on the relationship between children’s behaviors and parents’ private-domain sustainable behaviors. Compared to intragenerational caregiving, the mediating effect of environmental responsibility is more significant in the context of intergenerational caregiving.

*H7b:* Caregiver type moderates the mediating effect of environmental responsibility on the relationship between children’s behaviors and parents’ public-domain sustainable behaviors. Compared to intragenerational caregiving, the mediating effect of environmental responsibility is more significant in the context of intergenerational caregiving.

## 3 Research design and findings

This study systematically examined the mechanisms and moderating strategies of how children’s unsustainable behaviors influence parents’ sustainable behaviors through four formal experiments. Specifically:

Experiment 1 focused on the exposure effect of children’s unsustainable behaviors. By manipulating the actor (i.e., one’s own child vs. a neighbor’s child vs. no behavior), the study investigated whether parents are more inclined to engage in sustainable behaviors after being exposed to their own child’s unsustainable behaviors. The results revealed that when parents observed their own child’s unsustainable behaviors, their intentions to engage in both private-domain and public-domain sustainable behaviors significantly increased.

Experiment 2 examined the mediating roles of family responsibility and environmental responsibility. Using the same behavioral manipulation as in Experiment 1, with an additional no-behavior group for comparison, the results indicated that both family responsibility and environmental responsibility significantly mediated the positive impact of children’s unsustainable behaviors on parents’ private-domain sustainable behaviors. Additionally, environmental responsibility significantly mediated the positive impact of children’s unsustainable behaviors on parents’ public-domain sustainable behaviors.

Experiment 3 introduced caregiver type (intragenerational caregiving vs. intergenerational caregiving) as a moderating variable to explore its role in the relationship between children’s unsustainable behaviors and parents’ sustainable behaviors. The findings demonstrated that in intergenerational caregiving contexts, family responsibility and environmental responsibility significantly strengthened their mediating effects on the relationship between children’s unsustainable behaviors and parents’ private-domain sustainable behaviors. Similarly, environmental responsibility significantly enhanced its mediating effect on parents’ public-domain sustainable behaviors. These results suggest that familial closeness plays a critical moderating role in this mechanism.

Experiment 4 aimed to replicate Experiment 3 while addressing potential demand effects observed in Experiments 2 and 3. By applying different experimental contexts and designs, this study further validated the robustness of the findings.

Across these four experiments, the study tested the generalizability of the interactive model of sustainable behaviors within families from various situational and variable perspectives. The findings systematically revealed the mechanisms and pathways through which family members influence each other’s behaviors.

### 3.1 Experiment 1: main effects test

This study aimed to test H1, which posits that parents exposed to their own child’s unsustainable behavior, rather than the behavior of others with whom they have less close relationships (e.g., a neighbor’s child), would exhibit higher intentions to engage in sustainable behaviors.

#### 3.1.1 Experimental design

This study employed a 3 (behavior: own child’s unsustainable behavior, neighbor’s child’s unsustainable behavior, no behavior) between-subjects design, with all participants randomly assigned to one of the three groups. Sample size calculations were performed using G*Power 3.1 software, employing a one-way ANOVA, with an effect size (f) of 0.4, a significance level (*α*) of 0.05, a statistical power of 0.99, and three groups. The calculation indicated that a sample size of 138 participants was required. Ultimately, the study recruited 200 parents with children under the age of 18, and 179 valid questionnaires were returned, including 65 male and 114 female participants. In terms of age distribution, 53.3% of participants were between 20 and 40 years old. The sample primarily consisted of mothers, reflecting the characteristics of Chinese households with children.

#### 3.1.2 Questionnaire design and variable measurement

The experimental questionnaire consisted of four parts, as detailed below:

The first part was the informed consent form, which informed participants about the primary purpose of the questionnaire, clarified that the study was conducted solely for academic purposes, and assured participants that no personal privacy would be involved.

The second part was the “reading task,” designed to activate participants’ perceptions of children’s unsustainable behaviors. Participants were asked to read a scenario that manipulated the behavior of either “their own child” or “a neighbor’s child.” In the experimental context, participants read about their (or their neighbor’s) child eating at the dining table. The child could freely choose their preferred foods, filled their plate with food but only ate a small portion, leaving most of it wasted and dirty. Some unwanted food was pushed aside or even thrown onto the table or the floor. The child showed no concern for wasting food and continued to demand more food without finishing what was already on their plate. Following this task, participants responded to two manipulation check items: “I think my child’s behavior is unsustainable” and “I think the neighbor’s child’s behavior is unsustainable.” Higher scores indicated stronger perceptions of children’s unsustainable behaviors. The control condition asked participants to imagine “cleaning their house over the weekend.”

The third part measured sustainable behaviors. Participants completed a sustainable behavior scale, reporting the frequency with which they intended to engage in 12 sustainable behaviors over the next 3 months. These 12 behaviors included 7 private-domain sustainable behaviors and 5 public-domain sustainable behaviors. The items were adapted from Eom et al. (2018), Sparks et al. (2021), Cakanlar et al. (2023). Private-domain sustainable behaviors comprised 7 items (*α* = 0.842), such as: “using reusable shopping bags,” “unplugging or turning off electronic devices when not in use,” “conserving household water and electricity,” “reducing food waste,” “purchasing eco-friendly and energy-efficient products,” “reducing driving by walking, biking, or using public transportation,” and “recycling paper, plastic, and metal. “Public-domain sustainable behaviors comprised 5 items (*α* = 0.875), such as: “sharing environmental posts on social media,” “responding to environmental issues (online or offline),” “participating in environmental activities (e.g., trash collection),” “discussing environmental issues with others in the community,” and “actively participating in local environmental groups. “All items were measured using a five-point Likert scale (1 = “Never,” 2 = “Rarely,” 3 = “Sometimes,” 4 = “Often,” 5 = “Always”). The 12 behaviors were presented in a randomized order to prevent potential biases from systematic arrangement.

The fourth part collected demographic information.

#### 3.1.3 Results

First, a manipulation check was conducted on participants’ perceptions of children’s unsustainable behaviors. The mean score for perceiving the unsustainable behavior of one’s child in the group exposed to their own child’s unsustainable behavior was 4.84, while the mean score in the group exposed to the unsustainable behavior of a neighbor’s child was 4.45. The results indicate that participants in both groups were able to perceive children’s unsustainable behaviors, confirming the success of the independent variable manipulation.

Second, the main effects of children’s unsustainable behaviors on parents’ sustainable behaviors were examined. Results from ANOVA showed that, compared to those exposed to the unsustainable behavior of neighborhood children (M_neighbor_ = 3.62) and the control group (M_control_ = 3.21), those exposed to their own child’s unsustainable behaviors scored significantly higher on private-domain sustainable behaviors (M_own_ = 4.25; *F* (1,177) =5.56, *p* = 0.019). Similarly, compared to those exposed to the unsustainable behavior of neighborhood children (M_neighbor_ = 3.29) and the control group (M_control_ = 3.03), those exposed to their own child’s unsustainable behaviors scored significantly higher on public-domain sustainable behaviors [M_own_ = 4.39; *F* (1, 177) = 9.06, *p* = 0.003].

Third, chi-square tests were performed using children’s behavior as the row variable and gender, education level, age, and income as column variables. The results revealed no significant differences in the distribution of parents’ gender, education level, age, number of children, or income across different behavior groups.

Finally, an ANCOVA was conducted, with parents’ gender, education level, age, number of children, and income as covariates. The results revealed significant main effects of exposure to own child’s unsustainable behaviors (vs. neighbor’s child’s unsustainable behaviors) on parents’ private-domain sustainable behaviors [*F* (1, 177) = 44.85, *p* = 0.000], supporting H1a. Similarly, significant main effects were found for public-domain sustainable behaviors [*F* (1, 177) = 161.84, *p* = 0.000], supporting H1b. Additionally, ANCOVA results indicated that gender, age, number of children, and income did not produce significant main effects on either private-domain or public-domain sustainable behaviors.

In conclusion, this study validated the significant main effects of children’s unsustainable behaviors on parents’ sustainable behaviors using an experimental manipulation approach. Study 2 will further explore the mechanisms underlying these main effects.

### 3.2 Experiment 2: testing the mediating role of family responsibility and environmental responsibility

This experiment aimed to examine the mediating roles of family responsibility and environmental responsibility in the relationship between parents’ exposure to children’s unsustainable behaviors and their own sustainable behaviors (H2a, H2b, H3a, H3b). We hypothesize that, compared to the control group, parents exposed to their own child’s unsustainable behaviors will experience significantly heightened family responsibility and environmental responsibility, which in turn will enhance their engagement in sustainable behaviors.

#### 3.2.1 Experimental design

This study employed a laboratory experiment to further validate the main effects as well as the mediating roles of family responsibility and environmental responsibility. Sample size calculations were performed using G*Power 3.1 software, employing a one-way ANOVA, with an effect size (f) of 0.4, a significance level (*α*) of 0.05, a statistical power of 0.99, and two groups. The calculation indicated that a sample size of 118 participants was required. The study collaborated with the “Credamo” platform, randomly selecting participants from its database of families with children under the age of 18 on their platform. Participants were randomly assigned to one of two experimental groups: the “own child’s unsustainable behavior” group or the “control/no behavior” group. A total of 300 samples were collected, of which 29 invalid responses (e.g., responses completed too quickly or with invalid answers) were excluded, resulting in 271 valid responses. Among the valid samples, 70.5% of participants were female, and 53.7% were aged between 20 and 40 years.

#### 3.2.2 Questionnaire design and variable measurement

The experimental questionnaire consisted of five parts, as detailed below:

The first part was the informed consent form, which explained the purpose of the questionnaire to participants, clarified that the study was conducted solely for academic research, and assured participants that no personal privacy would be involved.

The second part was the “reading task,” designed to manipulate participants’ perceptions of their own child’s unsustainable behavior. This section followed the same design as Experiment 1. Participants were asked to read specific scenarios designed to manipulate perceptions of “their own child’s unsustainable behavior.” For the control group, the same neutral control condition information from Study 1 was provided (exposure to own child’s unsustainable behavior coded as 0; control group coded as 1).

The third part measured family responsibility and environmental responsibility. The family responsibility scale was adapted from Robinson et al. (1995), Smith and Williams (2010), and Sparks et al. (2021). It consisted of the following three items: “I have a responsibility to educate and guide the children in my family,” “I actively participate in my child’s learning and growth by providing necessary guidance,” and “I take primary responsibility for educating my child” (*α* = 0.838). The environmental responsibility scale was adapted from a scale developed by Dempsey and Sandler (1995) and Stone et al. (1995). and consisted of the following four items: “I am responsible for doing my part to protect the environment and conserve resources,” “I actively seek to learn about environmental protection,” “Even if my impact is small, I am committed to contributing to environmental protection,” and “My actions have an impact on the natural environment” (*α* = 0.810). Both scales were evaluated using a five-point Likert scale ranging from 1 (“strongly disagree”) to 5 (“strongly agree”).

The fourth part measured sustainable behaviors. This section was consistent with Experiment 1, requiring participants to complete a sustainable behavior scale that assessed the frequency of 12 sustainable behaviors they intended to perform over the next 3 months. The scale included 7 private-domain sustainable behaviors (*α* = 0.810), such as “using reusable shopping bags,” “conserving energy,” and “reducing food waste”; and 5 public-domain sustainable behaviors (*α* = 0.764), such as “participating in environmental activities” and “sharing environment-related content.” The 12 items were randomized during testing to avoid biases caused by systematic arrangement.

The fifth part collected demographic information.

#### 3.2.3 Results

First, the main effects of exposure to “own child’s unsustainable behavior” on parents’ sustainable behaviors were examined. ANOVA results revealed that participants in the “own child’s unsustainable behavior” group scored significantly higher on private-domain sustainable behaviors (M_0_ = 4.11) compared to those in the “control” group [M_1_ = 3.73; *F* (1, 269) = 21.364, *p* = 0.000]. Similarly, participants in the “own child’s unsustainable behavior” group scored significantly higher on public-domain sustainable behaviors (M_0_ = 4.49) compared to those in the “control” group [M_1_ = 4.29; *F* (1, 269) =11.927, *p* = 0.001].

Next, Chi-square tests were conducted using “own child’s unsustainable behavior” (“control”) as the row variable and gender, education level, and monthly income as column variables. The results indicated no significant differences in the distribution of gender, age, education level, number of children, or income across experimental groups.

Subsequently, an ANCOVA was conducted with gender, education level, age, number of children, and income as covariates. The results showed significant main effects of exposure to “own child’s unsustainable behavior” (“control”) on private-domain sustainable behaviors [*F* (1, 269) =3.922, *p* = 0.001] and public-domain sustainable behaviors [*F* (1, 269) =2.402, *p* = 0.028]. Moreover, the analysis revealed no significant main effects of parents’ gender, age, education level, number of children, or income on private-domain or public-domain sustainable behaviors. These findings further validated hypotheses H1a and H1b.

**Figure 1:**
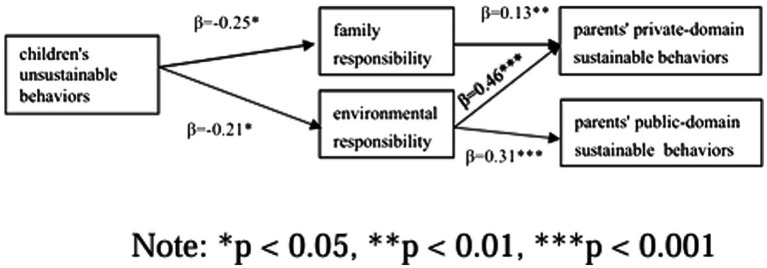
Mediating effects of family responsibility and environmental responsibility.

Finally, the mediating roles of family responsibility and environmental responsibility were examined using PROCESS 4.2 (Model 4, Bootstrapping 5,000 iterations), with gender, age, education level, number of children, and income included as covariates. The analysis yielded the following results (Figure 1):

*Private-domain sustainable behaviors*: Children’s unsustainable behavior had a significant negative effect on family responsibility (b = −0.25, SE = 0.10, t = −2.54, *p* = 0.011) and environmental responsibility (b = −0.21, SE = 0.09, t = −2.33, *p* = 0.020). Family responsibility significantly influenced private-domain sustainable behaviors (b = 0.13, SE = 0.05, t = 2.89, *p* = 0.004), as did environmental responsibility (b = 0.46, SE = 0.05, t = 9.03, *p* = 0.000). The 95% confidence intervals for the direct effect [−0.2245, −0.0390], the indirect effect of family responsibility [−0.0774, −0.0031], and the indirect effect of environmental responsibility [−0.1845, −0.0121] did not include 0. These results supported hypotheses H2a and H2b, indicating that children’s unsustainable behaviors negatively influenced parents’ private-domain sustainable behaviors directly and indirectly through family and environmental responsibility.

*Public-domain sustainable behaviors*: Family responsibility had no significant effect on public-domain sustainable behaviors (b = 0.03, SE = 0.03, t = 0.93, *p* = 0.352), whereas environmental responsibility had a significant positive effect (b = 0.31, SE = 0.03, t = 8.72, *p* = 0.000). The 95% confidence interval for the direct effect [−0.1366, −0.0129] and the indirect effect of environmental responsibility [−0.1250, −0.0098] did not include 0, but the confidence interval for the indirect effect of family responsibility [−0.0255, 0.0087] did include 0. These results supported hypotheses H3a and H3b, demonstrating that children’s unsustainable behaviors negatively influenced parents’ public-domain sustainable behaviors directly and indirectly through environmental responsibility, while family responsibility did not mediate this relationship significantly.

This study demonstrated that family responsibility and environmental responsibility played partial mediating roles in the relationship between children’s unsustainable behaviors and parents’ sustainable behaviors. Environmental responsibility significantly mediated both private-domain and public-domain sustainable behaviors, whereas family responsibility only mediated private-domain sustainable behaviors.

### 3.3 Experiment 3: testing the moderating role of caregiver type

This experiment aimed to investigate the moderating effect of caregiver type on the relationship between children’s unsustainable behaviors and parents’ sustainable behaviors. Based on our hypothesis, we propose that compared to the baseline condition (i.e., not being exposed to their own child’s unsustainable behaviors), when children are raised in an intergenerational caregiving context, parents exposed to their own child’s unsustainable behaviors will exhibit stronger family responsibility and environmental responsibility, which, in turn, will enhance their intention to engage in sustainable behaviors.

#### 3.3.1 Experimental design

This experiment employed a 2 (caregiver type: intergenerational caregiving vs. parent caregiving) × 2 (child’s unsustainable behavior vs. control/no behavior) between-subjects design. The required sample size was calculated using G*Power 3.1, with a one-way ANOVA test. The effect size (f) was set at 0.4, the significance level at 0.05, and the statistical power at 0.99, with four groups. The calculation indicated a required sample size of 152 participants. Ultimately, 220 parents with children under the age of 18 were randomly recruited via the “Credamo” platform. After excluding invalid responses, a total of 212 valid samples were collected (139 females and 73 males, the proportion of those aged 20–40 is 55.1%), and participants were randomly assigned to one of the experimental groups.

#### 3.3.2 Questionnaire design and variable measurement

The experimental questionnaire consisted of six sections, detailed as follows:

The first section was the informed consent form, which explained the purpose of the questionnaire to participants, clarified that the study was for academic research only, and assured participants that no personal privacy would be involved.

The second section manipulated the perception of caregiver type.

In the intergenerational caregiving group, participants were asked to recall and describe a situation in which their child was primarily cared for by grandparents. For example: “During this time, was your child mainly cared for by their grandparents? Please recall the interactions between your child and the grandparents, the behavior of the grandparents during caregiving, and your feelings about this arrangement.” This could include scenarios such as: “Did your child spend holidays, weekends, or times when you were busy being cared for by their grandparents? Please describe the interactions between your child and the grandparents and your perceptions and feelings about this caregiving arrangement.”

In the parent caregiving group, participants were asked to recall and describe a time they spent with their child. For example: “During this time, what interactions did you have with your child? How did you participate in your child’s growth process?” This could include specific examples such as: “Helping your child with homework, teaching them life skills, spending holidays together, or sharing other significant moments.” Participants were also asked to describe their feelings during these moments and their perceived role in their child’s development.

The third section was a “reading” task designed to manipulate the perception of the child’s unsustainable behavior. In Experiment 3, the scenario depicted a child wasting energy. Participants were asked to read the following scenario:

“Your child is playing in the living room, using colored paper, plastic bottles, and old cardboard boxes to create crafts. During this activity, the child turns on all the lights in the house and does not turn them off even when sunlight is streaming in. When using glue, the child opens the bottle cap and accidentally spills excess glue, most of which is wasted. After completing the craft, the child runs to the faucet, turns on the water to rinse their hands, and leaves the water running even after drying their hands, until an adult comes by to turn it off.” The no-behavior control group was asked to imagine themselves cleaning their home on a weekend. Exposure to own child’s unsustainable behavior coded as 0; control group coded as 1.

The fourth section measured sustainable behavior intentions, consistent with Experiment 2. Participants indicated their intentions to engage in private-domain sustainable behaviors (*α* = 0.853) and public-domain sustainable behaviors (*α* = 0.897) over the next 3 months.

The fifth section measured family responsibility and environmental responsibility, consistent with Experiment 2. The reliability coefficients for the family responsibility scale and environmental responsibility scale were *α* = 0.903 and *α* = 0.839, respectively.

The sixth section collected demographic information.

#### 3.3.3 Results

**Figure 2:**
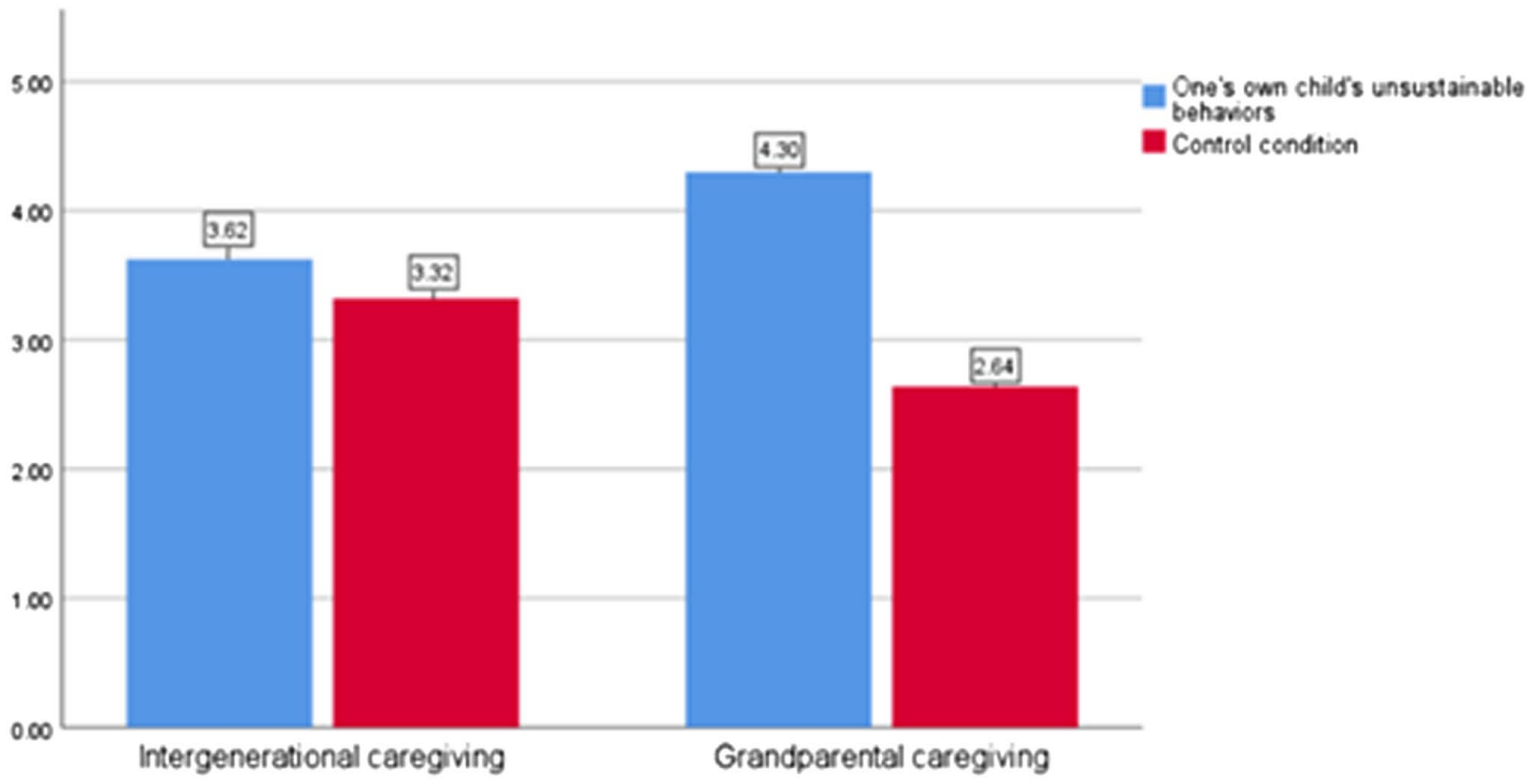
Parents’ family responsibility under different caregiver types (Study 3).

Interaction effect analysis revealed a significant interaction between children’s behaviors and caregiver type (*F* = −5.606, *p* = 0.00). As shown in Figure 2, under the parent caregiving condition, no significant difference was observed in parents’ family responsibility across experimental groups [M_0_ = 3.62, M_1_ = 3.32; *t* (1, 210) =1.436, *p* = 0.157]. However, under the intergenerational caregiving conditions, children’s behaviors had a significant main effect on parents’ family responsibility [M_0_ = 4.3, M_1_ = 2.64; *t* (1, 210) =12.702, *p* = 0.00]. Therefore, H4 was supported.

**Figure 3:**
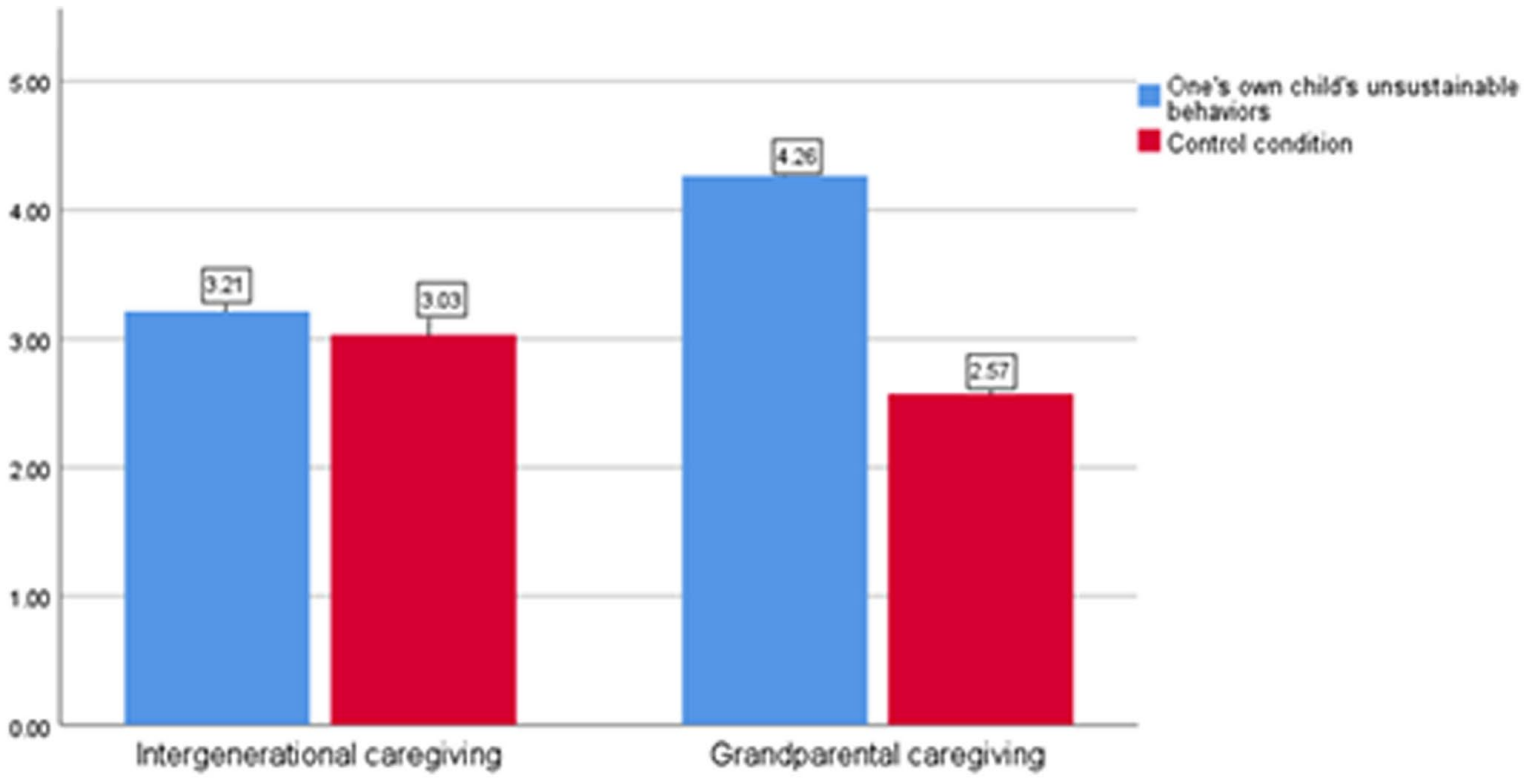
Parents’ environmental responsibility under different caregiver types (Study 3).

The interaction analysis with environmental responsibility as the dependent variable revealed a significant interaction between children’s behaviors and caregiver type [*t* (1, 210) = −5.871, *p* = 0.00]. As shown in Figure 3, under the parent caregiving condition, no significant differences were observed in parents’ environmental responsibility across the experimental groups [M_0_ = 3.21, M_1_ = 3.03; *t* (1, 210) = 0.693, *p* = 0.492]. However, under the intergenerational caregiving conditions, children’s behaviors had a significant main effect on parents’ environmental responsibility [M_0_ = 4.26, M_1_ = 2.57; *t* (1, 210) = 8.900, *p* = 0.00]. Based on these findings, H6 was supported.

This study applied PROCESS Model 8 to examine the moderating role of caregiver type and the moderated mediation effects of family responsibility and environmental responsibility in the proposed model. For private sustainable behavior, the analysis revealed a significant interaction between caregiver type and children’s behaviors (t = −5.740, *p* = 0.000). In the parent caregiving group, the mediation effect of family responsibility was not significant (*β* = −0.086, SE = 0.064, 95% CI [−0.224, 0.031]); however, in the intergenerational caregiving, family responsibility significantly mediated the effect of children’s behaviors on parents’ private sustainable behavior (*β* = −0.476, SE = 0.110, 95% CI [−0.671, −0.258]). Additionally, the index of moderated mediation for family responsibility was significant (*β* = −0.390, SE = 0.110, 95% CI [−0.600, −0.193]), indicating that family responsibility served as a significant moderated mediator in the model, supporting H5a.

In the parent caregiving group, the mediation effect of environmental responsibility was not significant (*β* = −0.047, SE = 0.075, 95% CI [−0.201, 0.096]); however, in the intergenerational caregiving group, environmental responsibility significantly mediated the effect of children’s behaviors on parents’ private sustainable behavior (*β* = −0.477, SE = 0.089, 95% CI [−0.641, −0.294]). Furthermore, the index of moderated mediation for environmental responsibility was significant (*β* = −0.430, SE = 0.115, 95% CI [−0.679, −0.225]), confirming that environmental responsibility acted as a significant moderate mediator in the model, supporting H5b.

For public sustainable behavior, the results also indicated a significant interaction between caregiver type and children’s behaviors (t = −5.483, *p* = 0.000). In the parent caregiving group, the mediation effect of family responsibility was not significant (*β* = −0.026, SE = 0.028, 95% CI [−0.094, 0.017]); similarly, in the intergenerational caregiving group, family responsibility did not significantly mediate the effect of children’s behaviors on parents’ public sustainable behavior (*β* = −0.142, SE = 0.095, 95% CI [−0.327, 0.047]). Additionally, the index of moderated mediation for family responsibility was not significant (*β* = 0.112, SE = 0.081, 95% CI [−0.281, 0.042]), indicating that family responsibility was not a significant moderated mediator in the model, and H7a was not supported. This result aligns with findings from Experiment 2, which also demonstrated that family responsibility did not have a significant relationship with public sustainable behavior.

In the parent caregiving group, the mediation effect of environmental responsibility was not significant (*β* = −0.043, SE = 0.068, 95% CI [−0.184, 0.085]); however, in the intergenerational caregiving group, environmental responsibility significantly mediated the effect of children’s behaviors on parents’ public sustainable behavior (*β* = −0.423, SE = 0.092, 95% CI [−0.606, −0.247]). Additionally, the index of moderated mediation for environmental responsibility was significant (*β* = −0.390, SE = 0.103, 95% CI [−0.600, −0.199]), indicating that environmental responsibility acted as a significant moderated mediator in the model, supporting H7b.

Across all tests, demographic covariates such as parents’ gender, age, education level, number of children, and income had no significant main effects on either private or public sustainable behaviors.

### 3.4 Experiment 4: replicating study 3 (addressing demand effects)

#### 3.4.1 Experimental design

In Experiment 4, we replicated the findings from Study 3. Since the measurement of the dependent variable followed the independent variable in Study 3, this design may have inadvertently introduced demand effects (i.e., participants reporting greater intentions for sustainable behavior might have also perceived themselves as having stronger responsibilities). To address this issue, Experiment 4 adjusted the experimental design by balancing the measurement order of the mediators and dependent variables and by measuring, rather than manipulating, caregiver type.

This experiment employed a two-level between-subjects design (children’s unsustainable behavior vs. control/no behavior). The required sample size was calculated using G*Power 3.1 software with a one-way ANOVA model. An effect size (f) of 0.4, a significance level of 0.05, a statistical power of 0.99, and two group were specified, resulting in an estimated sample size of 118. Ultimately, 260 parents were randomly recruited for the study, and after excluding invalid responses, a total of 244 valid samples were obtained (male n = 94, the proportion of the population aged 20–40 is 57.6%). Participants were randomly assigned to experimental groups.

#### 3.4.2 Questionnaire design and variable measurement

The experimental questionnaire consisted of six sections.

In the first section, participants were informed about the purpose and content of the questionnaire. It was clearly stated that the study was for academic research purposes only and did not involve the collection of personal information.

In the second section, participants were randomly assigned to read scenarios from Experiment 2 or Experiment 3. These scenarios described either “their own (or a neighbor’s) child wasting food” or “not engaging in recycling.” Participants in the control group were asked to imagine themselves cleaning their house on a weekend.

The third section involved completing scales measuring family responsibility (Cronbach’s *α* = 0.870) and environmental responsibility (Cronbach’s *α* = 0.877), consistent with the procedures used in Experiments 2 and 3.

In the fourth section, participants were asked to identify the primary caregiver of their child, choosing between intergenerational caregiving or parents.

The fifth section required participants to report their intentions to engage in private sustainable behaviors (Cronbach’s *α* = 0.845) and public sustainable behaviors (Cronbach’s *α* = 0.881) over the next 3 months, following the same methodology as Experiment 2.

The final section collected demographic information.

#### 3.4.3 Results

**Figure 4:**
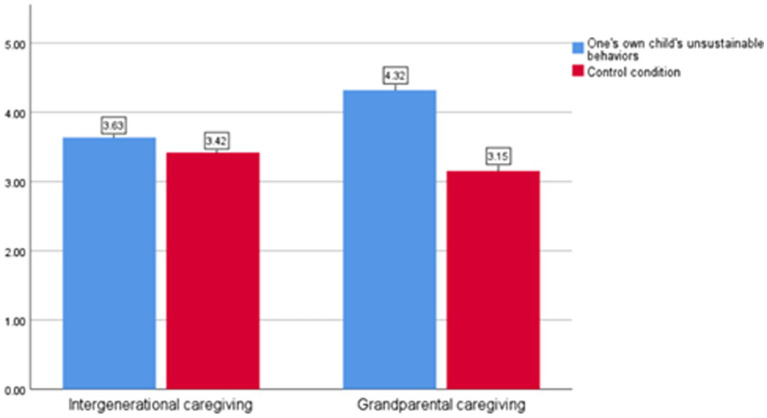
Parents’ family responsibility under different caregiver types (Study 4).

The results demonstrated a significant interaction effect between children’s behavior and caregiver type (*F* = 21.737, *p* = 0.00). As shown in Figure 4, under parental caregiving conditions, there was no significant difference in family responsibility scores between participants in different groups [M₀ = 3.63, M₁ = 3.42; *t* (1, 222) = 1.441, *p* = 0.152]. However, under intergenerational caregiving conditions, the main effect of children’s behavior on family responsibility was significant [M₀ = 4.32, M₁ = 3.15; *t* (1, 222) = 8.195, *p* = 0.00]. These findings provide further support for H4.

**Figure 5:**
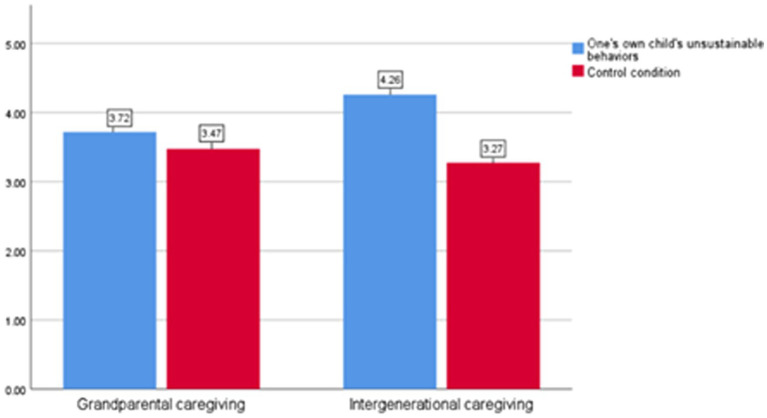
Parents’ environmental responsibility under different caregiver types (Study 4).

The moderating effect of caregiver type on the relationship between children’s behavior and environmental responsibility was tested. The results indicated a significant interaction effect between children’s behavior and caregiver type (*F* = 14.365, *p* = 0.00). As shown in Figure 5, under parental caregiving conditions, there was no significant difference in environmental responsibility scores between participants in different groups [M_0_ = 3.72, M_1_ = 3.47; *t* (210) = 1.690, *p* = 0.094]. However, under intergenerational caregiving conditions, children’s behavior had a significant main effect on parents’ environmental responsibility [M_0_ = 4.26, M_1_ = 3.27; *t* (222) = 6.750, *p* = 0.00]. These findings provide further confirmation of H6.

The analysis of intentions to engage in private-domain sustainable behaviors yielded the following results. In the parental caregiving group, the mediating effect of family responsibility was not significant (*β* = −0.034, SE = 0.032, 95% CI [−0.110, 0.014]). However, in the intergenerational caregiving group, family responsibility showed a significant mediating effect on the relationship between children’s behavior and parents’ private-domain sustainable behaviors (*β* = −0.200, SE = 0.080, 95% CI [−0.367, −0.052]). Additionally, the moderated mediation index for family responsibility was significant (*β* = −0.166, SE = 0.070, 95% CI [−0.318, −0.041]), indicating that family responsibility serves as a significant moderated mediator in the research model, which further supports Hypothesis H5a.

For environmental responsibility, the mediating effect was not significant in the parental caregiving group (*β* = −0.107, SE = 0.073, 95% CI [−0.267, 0.018]). However, in the intergenerational caregiving group, environmental responsibility had a significant mediating effect on the relationship between children’s behavior and parents’ private-domain sustainable behaviors (*β* = −0.473, SE = 0.103, 95% CI [−0.688, −0.286]). The moderated mediation index for environmental responsibility was also significant (*β* = −0.4735, SE = 0.103, 95% CI [−0.688, −0.286]), demonstrating that environmental responsibility is a significant moderated mediator in the research model, thereby supporting Hypothesis H5b.

For public-domain sustainable behaviors, the results indicated that the interaction between caregiver type and children’s behavior was significant (*t* = −4.78, *p* = 0.000). In the parental caregiving group, the mediating effect of family responsibility was not significant (*β* = −0.021, SE = 0.022, 95% CI [−0.075, 0.148]). Similarly, in the intergenerational caregiving group, family responsibility did not show a significant mediating effect on the relationship between children’s behavior and parents’ public-domain sustainable behaviors (*β* = −0.123, SE = 0.083, 95% CI [−0.292, 0.031]). Furthermore, the moderated mediation index for family responsibility was not significant (*β* = −0.102, SE = 0.074, 95% CI [−0.263, 0.025]), indicating that family responsibility is not a significant moderated mediator in the research model, which corroborates the non-significance of Hypothesis H7a.

For environmental responsibility, the mediating effect was not significant in the parental caregiving group (*β* = −0.068, SE = 0.045, 95% CI [−0.164, 0.011]). However, in the intergenerational caregiving group, environmental responsibility demonstrated a significant mediating effect on the relationship between children’s behavior and parents’ public-domain sustainable behaviors (*β* = −0.299, SE = 0.086, 95% CI [−0.478, −0.141]). The moderated mediation index for environmental responsibility was also significant (*β* = −0.231, SE = 0.082, 95% CI [−0.409, −0.091]), establishing that environmental responsibility is a significant moderated mediator in the research model, thus supporting Hypothesis H7b.

Across all analyses, the covariates (gender, age, education, number of children, and income) of parents did not exhibit significant main effects on either private-domain or public-domain sustainable behaviors.

## 4 Research conclusions and implications

### 4.1 Research conclusions

This study systematically explored the interactive patterns of sustainable behaviors among children, parents, and grandparents within families, focusing on the impact of children’s unsustainable behaviors on parental sustainable behaviors and the moderating role of caregiver types. The findings indicate that children’s unsustainable behaviors significantly influence parental decisions regarding sustainable behaviors. Specifically, when parents are exposed to their children’s unsustainable behaviors, their intentions to engage in both private and public sustainable behaviors are significantly enhanced.

Furthermore, this study revealed the mediating roles of family responsibility and environmental responsibility in the relationship between children’s unsustainable behaviors and parental sustainable behaviors. The results show that, when confronted with their children’s unsustainable behaviors, parents tend to exhibit stronger feelings of family responsibility and environmental responsibility. These emotions drive an increase in parents’ private sustainable behaviors, while a higher sense of environmental responsibility further promotes their public sustainable behaviors. The research findings indicate that the mediating effect of family responsibility on parents’ pro-environmental behavior in the public domain is not significant. This is primarily because pro-environmental behavior in the public domain extends beyond the family context, is less visible, and more covert, making it difficult to serve as an effective model in daily parenting practices.

In contexts of intergenerational caregiving, these effects are more pronounced. When grandparents serve as the primary caregivers, parental family responsibility and environmental responsibility are further reinforced, leading to a greater likelihood of parents engaging in private sustainable behaviors. Simultaneously, enhanced environmental responsibility motivates parents to adopt more public sustainable behaviors, addressing the potential threats posed by children’s unsustainable behaviors to both family and environmental responsibilities. These findings provide a novel perspective for understanding the dynamic interaction mechanisms of sustainable behaviors within families and offer empirical evidence to support the development of family-oriented environmental education and behavioral intervention strategies.

### 4.2 Theoretical contributions

The theoretical contributions of this study are reflected in the following three aspects: First, the study offers a novel perspective on intergenerational sustainability behavior interactions. While existing research on intergenerational sustainability behavior interactions primarily focuses on the impacts of positive sustainable behaviors (Žukauskienė et al., 2020; Bulkeley and Gregson, 2009), this study explores the influence of unsustainable behaviors within the context of family interactions, thereby enhancing our understanding of the mechanisms of sustainable behavior interactions in the field of consumer behavior.

Second, in existing research, social norms and a sense of responsibility have been demonstrated to significantly influence individuals’ pro-environmental behaviors (Nolan, 2020). This study identifies the mediating roles of family responsibility and environmental responsibility, providing new empirical evidence for understanding the influence of family members on each other’s behaviors and their underlying mechanisms. This also offers a foundation for further exploration of emotion-driven sustainable behaviors.

Third, the study validates the moderating role of caregiver type as a boundary condition, shedding light on how family structure influences the transmission of sustainable behaviors. This finding aligns with previous research on the influence of social relationship strength on behavioral decision-making (Kim and Kochanska, 2020b; Maiya et al., 2022).This provides novel perspectives and empirical support for family behavior research within cultural and social contexts.

### 4.3 Managerial implications

Firstly, facilitating the development of family education strategies. This study examines how children’s unsustainable behaviors influence parents’ sustainable behaviors, creating a behavioral cycle within the family. Educational institutions and family educators can design more effective family education strategies. Families can address children’s unsustainable behaviors by reinforcing positive role models, thereby promoting sustainability within the household. For instance, parents can demonstrate environmental responsibility by participating in environmental protection activities or adopting energy-saving measures. Such practices help integrate environmental responsibility as a key component of family education.

Secondly, informing corporate marketing strategies. The study reveals the moderating role of caregiver type. For families with intergenerational caregivers, emphasizing shared family responsibilities and long-term environmental goals may resonate more strongly. In contrast, for nuclear families, offering personalized and easily implementable sustainable practices may increase the acceptance and usage of environmentally friendly products.

Finally, promoting societal sustainability. Policymakers and social organizations can enhance environmental education at the family level, driving societal transformation toward sustainability. For example, communities and schools could organize events such as “Parent–Child Environmental Actions” or “Family Environmental Responsibility” campaigns to encourage families to engage in environmental practices together. Strengthening environmental education at the family level can effectively guide family members to internalize sustainable behaviors as part of their daily routines.

### 4.4 Limitations and future directions

This study has several limitations, as outlined below. First, although this research focuses on the roles of family responsibility and environmental responsibility in the interaction of sustainable behaviors within families, other factors that may influence parents’ sustainable behaviors have not been considered. For instance, variables such as parents’ environmental awareness and self-efficacy traits might impact the findings, warranting further exploration in future research. Second, while this study confirms the moderating role of caregiver type, other potential moderating variables remain insufficiently examined. For example, cultural background, the gender of the child and communication patterns among family members might influence the results. Future research could delve deeper into these aspects to further refine the theoretical framework of family sustainable behavior interactions.

## Data Availability

The raw data supporting the conclusions of this article will be made available by the authors, without undue reservation.
